# The modular approach to therapy for youths with anxiety, depression, trauma, and conduct problems (MATCH): results from the Norwegian randomized-controlled trial

**DOI:** 10.1186/s40359-024-02082-0

**Published:** 2024-10-18

**Authors:** Silje S. Hukkelberg, Torbjørn Torsheim, Kristin Berg Nordahl, Görel E. Bringedal, Sivarajan Rajah, Kristine Amlund Hagen, John Kjøbli, Kristian Rognstad, Ana M. Ugueto, Sarah Kate Bearman, John Weisz

**Affiliations:** 1 The Norwegian Center for Child Behavioral Development (NCCBD), P.O. Box 7053, Majorstuen, Oslo, 0306 Norway; 2 Department of Psychosocial Science, University of Bergen, Bergen, 5020 Norway; 3 Regional Center for Child and Adolescent Mental Health, Eastern and Southern Norway, Postboks 4623, Nydalen, Oslo, 0405 Norway; 4 Texas Children’s Hospital, Department of Pediatrics, Baylor College of Medicine, 6701 Fannin St, Houston, TX 77030 USA; 5 Ballmer Institute for Children’s Behavioral Health, University of Oregon, 2800 NE Liberty St, Portland, , OR 97211 USA; 6 Department of Psychology, Faculty of Arts & Sciences, Harvard University, William James Hall, 33 Kirkland Street, Cambridge, MA USA

**Keywords:** Effectiveness study, Modular approach, Youths, Externalizing and internalizing problems, Evidence-based treatment, Adherence to therapy

## Abstract

**Background:**

A randomized controlled trial was conducted to examine the effectiveness of the Modular Approach to Therapy for Youths with Anxiety, Depression, Trauma, and Conduct Problems (MATCH) for Norwegian youths referred to seven Child and Adolescent Psychiatric Outpatient Clinics. MATCH addresses comorbid problems that are common in children and youth, and its transdiagnostic design may therefore be more effective compared to standard treatments that often address single problems. MATCH has, however, never been evaluated in a Nordic context, and the present study aimed to fill this gap.

**Methods:**

A sample of 121 Norwegian youths (*M*_age_ = 9.83, 58.7% boys) was randomly assigned to MATCH (*n* = 73) or treatment as usual (TAU, *n* = 48). Primary treatment outcomes were youths’ externalizing and internalizing problems as reported by parents, using the Child Behavior Checklist, the Behavior and Feelings Survey. In addition, the study included assessments of parent-reported Top Problems.

**Results:**

Overall, youths showed significant improvements in both externalizing and internalizing problems from intake to post-test. Results did not provide evidence that MATCH reduces symptoms of these problems compared to TAU.

**Conclusions:**

The findings were inconclusive regarding whether MATCH was more effective than TAU in reducing youth internalizing and externalizing problems.

**Trial Registration Identifier:**

ISRCTN24029895. Registration date: 8/8/2016.

**Supplementary Information:**

The online version contains supplementary material available at 10.1186/s40359-024-02082-0.

## Introduction

The prevalence of mental health issues among children and adolescents (hereafter “youths”) is a pressing matter [1, 2]. Notably, findings indicate that one in seven youths aged 10 to 19 years old suffers from at least one mental health disorder, which accounts for about 13% of the global burden of disease in this age group [3]. The number aligns with European statistics from Sacco et al. [4], reporting a 16% prevalence of mental health disorders among youths aged 5 to 18 years. Untreated mental health problems lead to substantial costs to individuals and to society, through e.g., disrupted education, impaired school performance, social exclusion, and exacerbation of diverse health challenges [5–8]. Hence, a strong case can be made for providing mental health care to young people, with a particular emphasis on evidence-based treatments (EBTs) that have been proven to have beneficial effects.

In Norway, about 8% of youths have been found to meet the criteria for a mental health disorder [9–11]. However, children’s rapid development can result in different expressions of mental health problems across ages [12], and symptoms across multiple areas. A population-based study among youths aged 8 to 10 years, indicates that 26% of all Norwegian youths with one mental health condition also suffered from another mental health condition [13]. This finding underscores the importance of identifying comorbid conditions in youths to ensure effective treatment responses and outcomes. In addition, it demonstrates the need for professionals to develop awareness and expertise in managing multiple mental health conditions at the same time. In Norwegian child and adolescent mental health clinics (CAMHS), some of the most common reasons for referral include depression, anxiety, conduct problems, and trauma-related difficulties [14]. Interventions used to address these mental health issues include Cool Kids for Anxiety, Depression Management for Youth [15] 2013), Trauma-Focused Cognitive Behavioral Therapy [TF-CBT] [16], and Parent Management Training, the Oregon Model [PMTO] [17, 18]. Each of these treatments are evidence-based with documented treatment effects in Norway [19–22]. However, typically, these programs focus on a single disorder, or a homogeneous cluster of symptoms, and encompass a predefined sequence of treatment content. This can present challenges for clinicians when treating youth with comorbid conditions or diverse needs.

The Modular Approach to Therapy for Children with Anxiety, Depression, Trauma, and Conduct Problems (MATCH) is a transdiagnostic US-developed treatment program [23] towards youths with co-occurring emotional and behavioural problems. MATCH uses evidence-based practices in the areas of anxiety, depression, trauma, and conduct problems by combining different treatment modules into a flexible and manualized treatment approach [24]. As such, MATCH is tailored to fit each youth’s specific needs at intake, and potential changes that may arise during treatment, by addressing common (e.g., emotional regulation) and disorder-specific treatment components (e.g., the “fear ladder” in treatment of anxiety).

Therapy starts out with a comprehensive assessment of a youth’s problems, strengths and needs, to identify appropriate treatment modules. Each module consists of techniques to address a specific problem and evidence-based strategies. After selecting the most relevant modules, treatment is delivered to address the needs of each youth individually. For example, treatment for anxiety may begin with the module on psychoeducation about anxiety, followed by cognitive restructuring, exposure exercises, and relaxation techniques to manage high levels of arousal. If anxiety is co-occurring with another mental health problem, such as depression or trauma, clinicians integrate modules that address these issues into the overall comprehensive treatment.

Recent findings suggests that MATCH may be an effective treatment approach for young people. A randomized-controlled effectiveness trial (RCT), conducted with a sample of 174 American clinically-referred youths aged 7–13 years, showed that MATCH outperformed treatment as usual (TAU), and standard EBT practice [25]. Specifically, youths in the MATCH group showed steeper slopes of improvement in internalizing and externalizing problems than youths receiving the other two treatments, from baseline to post-test. A follow-up assessment, two years after treatment was finished, showed sustained and superior effects for MATCH over TAU [26]. In a second study, among youths aged 5–15, MATCH showed a faster reduction in problem severity and improvement on functional outcomes, compared to multiple evidence-based practices [27]. On the other hand, the superior effects of MATCH were not reproduced in a recent US randomized controlled effectiveness trial among 156 youths ages 6 to 16 [28]. Neither were the results replicated in a more recent study from New Zealand [29], where MATCH was compared to usual care among 206 young participants (age 7–15). In summary, the evidence regarding the effectiveness of MATCH versus TAU is mixed. In addition, the question of whether the beneficial effects of MATCH translate to settings outside the United States remains largely unanswered.

While Norway and US share some similarities in the approach to mental health treatment, there are also notable differences that may influence treatment outcomes. First, geographically, Norway is a long country with low population densities in some regions. Thus, CAMHS in certain rural areas of Norway may have difficulties recruiting sufficient expertise and getting enough volume to gain experience in treating child mental health problems. On the other hand, because Norway has a universal health care system, the accessibility and delivery of health care services may facilitate earlier and more uniform diagnosis and treatment. Furthermore, different cultural contexts influence behavioral norms, help-seeking behavior, parental styles, and perceived stigma, all of which may influence potential outcomes of mental health services for youth. Finally, while the inclusion and exclusion criteria used in the present study are the same as those employed in the US trials of MATCH, we expect nonetheless that sample characteristics across countries will vary due to social, ethnic, economic, and genetic differences. Indeed, these are some of the most important reasons for testing whether the MATCH treatment effects can be transferred to other contexts and other populations.

## The present study

The aim of this study was to investigate the effectiveness of the MATCH intervention among Norwegian youths referred to seven different CAMHS. Many current treatment programs adopt a single approach or address homogenous clusters of symptoms and may not adequately address the diverse needs of youths with mental health problems. MATCH, as a flexible and modular approach, could therefore potentially enhance the effectiveness and efficiency of treatment.

In this study, we compared MATCH with treatment as usual (TAU) by conducting a multi-site randomized controlled trial (RCT) among Norwegian youths aged 6 to 14.5 years at the time of intake. Clinical outcomes were parent-reported youth externalizing and internalizing problems assessed at intake, end of treatment (post-test), and 12 months thereafter (follow-up). The study sought to answer two questions: [1] Do youths in the MATCH group have larger reductions in externalizing and internalizing problems from intake to post-test and follow-up compared to those assigned to TAU? and [2] Is MATCH more time-efficient compared to TAU?

## Method

### Design and procedure

The study was designed as a RCT with two conditions, MATCH and TAU, with 1:1 allocation. The trial was carried out by The Norwegian Center for Child Behavioral Development (NCCBD). The MATCH study was approved by the Regional Committee for Medical and Health Research Ethics (ID #28038), and the study was preregistered in ISRCTN, see 10.1186/ISRCTN24029895. The study protocol is published in Trials [30]. The study adheres to the CONSORT reporting guidelines [31].

The sample consisted of youths aged 6 to 14.5 years, who were referred to one of seven different Child and Adolescent Psychiatric Clinics (CAMHS) in Norway, from February 2015 to October 2020. The CAMHS are organized under the specialist healthcare services, and referrals are usually done by a physician, head of the municipal youth welfare agency, or a community psychologist. The seven clinics recruited families to be assessed for eligibility for the study. The research coordinators at NCCBD then contacted the families, who were informed about the study. Inclusion criteria were (a) youth age between 6 and 14.5 years at intake, (b) scores above clinical cut-off scores on internalizing or externalizing scales, or traumatic experiences/symptoms, (c) at least one of the parents must be able to understand the consent form and answer the questionnaires and interviews in Norwegian. Criteria for exclusion included youth psychosis, intellectual disability, pervasive developmental disorder, anorexia, bulimia, perpetrator of sexual assault, suicidality, or serious self-harms. Youths were excluded if problems were serious antisocial/criminal behaviour or substance abuse only (see 23 for more details). In addition, the youth could not receive active treatment simultaneously from another provider.

**Fig. 1:**
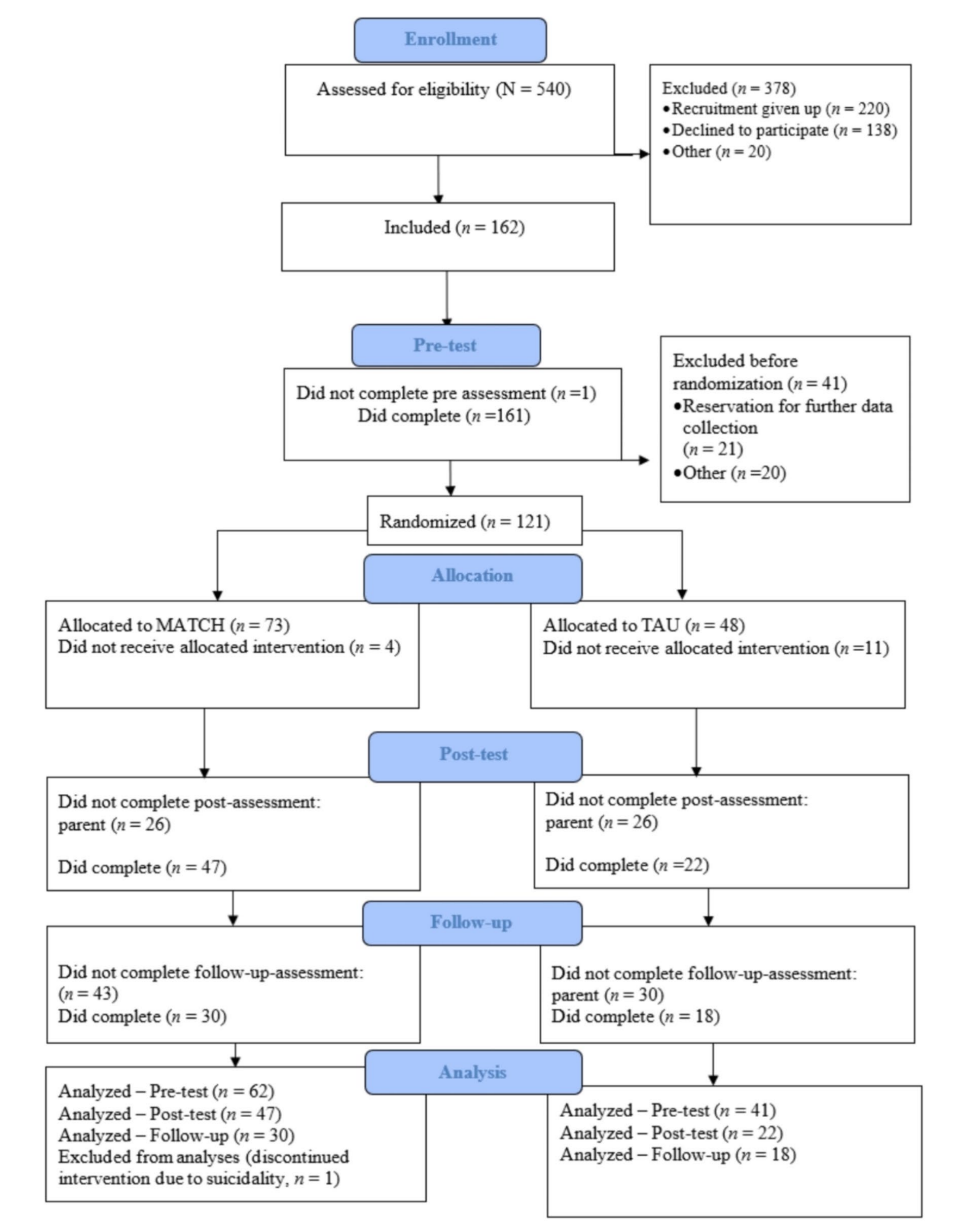
Flowchart of youth participants

Both parents provided written informed consent to the research coordinators. Thereafter, the youths were randomly assigned to either the MATCH or TAU condition. The allocation sequence was generated at random using the website www.random.org, and the research coordinators at NCCBD were responsible for the randomization process and oversaw its implementation. One sequence was generated for each treatment site (stratification variable). The researchers were blind to youths’ identification and allocation status. A total of 540 youths were screened for possible participation. Out of these, 162 youths (*n* = 71 boys) met the criteria for inclusion, but 121 youths were randomized to either of the conditions (see Fig. 1). Participating families received approximately $36 per completed survey, which was completed online at home or at the CAMH clinics.

### Participants

A total of 48 (39.7%) youths were allocated to TAU whereas 73 (60.3%) were allocated to MATCH. Mean age at intake was 9.83 (*SD* = 2.91, range = 6–14.5), and 71 (59.9%) were boys. At intake, Top Problems Assessment (TPA) showed that anxiety was most common (*n* = 51, 42.5%), followed by conduct problems (*n* = 36, 30.0%), depression (*n* = 15, 12.4%), and trauma (*n* = 12, 9.9%). Seven youths (5.8%) were missing. The youths with traumas reported on average 1.56 traumatic events (*SD* = 1.43, range: 0–6). Most youths lived in a family with married/cohabiting parents (*n* = 75, 62%). Mean age of parents was 40.34 (*SD* = 5.96), and most had education at university/college level (*n* = 64, 52.8%), while 47 (38.9%) had education at high-school level or lower. Mean family income before taxes was NOK 986,655 (*SD* = 778, 664, range: 0–8 mill), which reflects a typical median income level in a family with kids in Norway [32].

At intake, parents displayed relatively low levels of mental distress (i.e., anxiety and depression), as indicated by a Symptom Cheklist-5 (SCL-5 [33]) score of *M* = 1.77 (*SD* = 0.65, range 1-3.60), which falls below the cut-off value of 2.00 that is suggested as a predictor of mental distress (cf. 25). Table 1 shows characteristics of the study sample. As seen, the MATCH and TAU groups are comparable in terms of gender distribution, mean age, quality of life as reported by parents (Kidscreen-10), and problem areas.

**Table 1:** Sample characteristics at intake by treatment group

	MATCH	TAU	Group comparison
	*n* /Mean (SD)	*n* /Mean (SD)	χ^2^ test/ t-test
Youths (*n* = 121)	73 (60.3)	48 (39.7)	
Gender (boy/girl)	40/33	31/17	*p* = 0.363
Mean age	9.90 (SD = 2.32)	9.77, (SD = 1.97)	*p* = 0.747
Kidscreen-10	29.47 (SD = 5.58)	30.92 (SD = 5.45)	*p* = 0.485
Treatment areas			
Anxiety	33	18	
Conduct problems	20	16	
Depression/ trauma	18	9	*p* = 0.202

Note. Depression and trauma were grouped together due to small numbers (*n* ≤ 5)

### Therapists

A total of 47 therapists were recruited from seven different CAMHS in the Eastern and Western parts of Norway. Among these, 44 (94%) responded to questions mapping background characteristics and regular therapeutic practice. Responders stem from all seven clinics, the majority were females (*n* = 38 of 44, 86.4%) and had a Norwegian background (*n* = 41, 93.2%). Average age was 39 years (*SD* = 9.19), and mean years of clinical experience was 8 (*SD* = 6.11). Most therapists were clinical psychologists or physicians specialised in psychiatry (*n* = 27, 61.4%), 10 had a master’s degree (22.7%), and 6 had a bachelor/other type of education (13.6%). One did not respond to the question. Therapist background was unrelated to treatment group (*χ*2 = 0.125, *p* = 0.940).

Twenty MATCH therapists participated in a 5-day intensive training program provided by NCCBD free of charge. The training was conducted by experienced US MATCH experts, primarily led by S.K. Bearman and A.M. Ugueto, who presented the rationale and basic principles in MATCH, as well as the theoretical underpinnings and treatment procedures in the four treatment areas. All modules were presented in-depth and supported by modelling, video demonstrations from real cases, and role-playing. The therapists were taught to select and combine modules based on each youth’s needs, and in terms of progression throughout therapy in the format of on-the-job training with weekly “prep calls” with a Norwegian co-consultant and group consultations with US consultants (in English). The comparison therapists (*n* = 27) provided treatment as usual (TAU).

When a youth was randomized to a MATCH therapist, the therapist received information regarding the youth’s primary protocol, by a US consultant and a Norwegian co-consultant. The Norwegian MATCH study included an extra level of consultation, that is, Norwegian consultants were included together with the American ones, to address possible language barriers and ensure cultural adaptation to clinical practice. Table 2 provides a description of the therapists in both groups. As seen, there were no differences between the TAU and MATCH therapists in terms of mean age, years of clinical experience, years in present job, the usual number of patients simultaneously, and hours of guidance (usually discussions and reflections about academic challenges) each week. The therapists who delivered MATCH received 1-hour weekly net-based group consultations with a local co-consultant in addition to consultations with MATCH experts from the US (S. K. Bearman, E. Lee, H. MacPherson, L. Krumholz- Marchette, and A. M. Ugueto).

**Table 2:** Therapist characteristics at intake by treatment group

	MATCH	TAU	Group comparison
	*n* /Mean (SD)	*n* /Mean (SD)	*p*-value
N	20 (45.5%)	24 (54.5%)	
Age	40.40 (SD = 8.28)	37.13 (SD = 9.83)	*p* = 0.243
Years of clinical experience	9.30 (SD = 5.77)	6.87 (SD = 6.29)	*p* = 0.194
Years in present job	3.95 (SD = 3.08)	2.71 (SD = 2.90)	*p* = 0.183
Cases simultaneously	24.10 (SD = 6.55)	22.05 (SD = 5.35)	*p* = 0.285
Hours of weekly guidance	0.70 (SD = 0.73)	1.00 (SD = 0.92)	*p* = 0.261

Note. *n* = 44 (94%) therapists responded to the survey. P-values are based on t-tests

### Interventions

Therapists who are learning to implement MATCH, or part of trial, are encouraged to use three components: (1) the MATCH therapist manual [23], (2) a monitoring and feedback system for frequent assessment of each youth’s treatment response throughout care, and (3) weekly consultation with a MATCH expert/consultant. The monitoring and feedback system is used in clinical consultation with therapists to gauge, monitor progress, and guide treatment.

The MATCH treatment protocol brings together common and evidence-based treatment strategies for anxiety, depression, trauma and conduct problems in a modular and flexible system [25], and altogether 33 treatment modules are described. The Norwegian version of MATCH included two additional modules (“Learning about Traumatic Stress for Children” and “Learning about Traumatic Stress for Parents”), altogether 35 treatment strategies. Flowcharts provide help to therapists to individualizing treatment and advance across diagnosis and additional issues [34]. The MATCH approach thus makes it possible to combine several treatment protocols, and thereby address comorbid and fluctuating symptoms that may be present at intake or appear during therapy.

The youths received therapy on a weekly or bi-weekly basis, for about one hour at the clinic. Although parents were usually involved in treatment of youth conduct problems, they were also encouraged to participate in treatment of anxiety, depression, and trauma. Weekly, the youths (age ≥ 8) and parents answered the Progress Assessment in Therapy (PATH) questionnaire, which assessed and monitored treatment progress. This information was further used by the therapists and the consultant group to follow each patient’s progression in therapy, and if necessary, adjust and redefine the treatment goals.

Youths randomized to the TAU conditions received individual treatment, in accordance with each therapist’s usual practice. Most TAU-therapists reported substantial clinical experience (*M* = 6.87 years, *SD* = 6.29), and a significant number used a cognitive (*n* = 8) or eclectic approach (*n* = 4). There were no associations between treatment group and therapeutic approach (*χ*2 = 3.408, *p* = 0.333). As shown in Table 2, TAU-therapists reported that they received about 1 h of supervision per week, and that they had about 22 patients in treatment weekly.

### Adherence to treatment

To assess treatment adherence, we used the Adherence Coding Manual for MATCH.

 [35, 36], translated and adjusted to the Norwegian study [37] in collaboration with S.K. Bearman. Video-recorded therapy sessions were retrieved from 77 cases (64% of the total sample), 57 from the MATCH and 20 from the TAU condition. The assessments included micro-analytic coding of adherence to treatment i.e., if one or more of the 28 MATCH- specific therapist strategies derived from the 35 MATCH modules were used during treatment. The therapist strategies were coded in five-minute segments throughout full therapy sessions. Coding was done by a team of six coders (4 psychology students, 1 law student, and 1 copywriter) who were trained by one of the study researchers. To assess adherence to treatment, we used a stratified random sample of session recordings to ensure coverage of the four treatment aeras (anxiety, depression, trauma, and conduct problems). Altogether 468 treatment sessions were coded (358 from MATCH and 120 from TAU). A random sample of 37% (*n* = 174) of the recorded therapy sessions were double coded to assess inter-rater reliability. Overall reliability across strategies as measured by Cohen’s kappa was 0.76, which is regarded as adequate [38].

### Measures

In the following section, we provide brief descriptions of the measures used in this study. Comprehensive details regarding all the measures can be found in the study protocol [30].

### Primary outcomes

#### Child Behavior Checklist (CBCL)

The CBCL is part of the Achenbach System of Empirically Based Assessment (ASEBA [39]), , and was completed by parents to assess the youths’ externalizing and internalizing problems as well as associated sub-domains. We assessed five of the original seven symptom scales (i.e., aggressive behaviour, rule-breaking behaviour, somatic complaints, withdrawn/depressed, and anxiety/depression). Items were summarized into each sub-domain, and broadband scales from these were used to capture measures of internalizing (somatic complaints, withdrawn/depressed, and anxiety/depression) and externalizing problems (aggressive and rule-breaking behaviour). The CBCL was administered at intake, post-, and follow-up. Cronbach’s alpha reliability coefficients (α) were at intake/post/follow-up 0.878/0.916/0.889 for internalizing and 0.925/0.946/0.941 for externalizing problems (sub-domains ranged from 0.700/0.722/0.738 to 0.913/0.937/0.932).

#### Top problems Assessment (TPA)

Parents rated Top Problems Assessment (TPA [40, 41], to obtain severity ratings of the youth’s top three problems (from 0 to 4), identified in the pre-treatment interviews. Psychometric analyses of the TPA have shown strong reliability, validity, and sensitivity to change during treatment in earlier studies of MATCH in the US (Weisz, et al., 2012, Weisz et al., 2011). TPA were measured using the PATH system, and the ratings of the three problems were summarized to investigate the TPA total scores, at intake and end of treatment. Cronbach’s alphas for the TPA were 0.603 at intake and 0.796 at end of treatment.

#### Behavior and feelings Survey (BFS)

Parents filled out the Behavior and Feelings Survey (BFS; [42], a 12-item measure of internalizing (6 items; range from 0 to 12) and externalizing (6 items; range from 0 to 12) problems, in the PATH system at intake and end of treatment. The BFS was developed through psychometric testing of an item pool derived from the “Top Problems”, identified by previous samples of clinically referred youths and their parents. BFS scores have shown convergent and discriminant validity with other parent-report problem scales, including the CBCL. Cronbach’s alphas were 0.866 at intake and 0.917 at end of treatment for externalizing problems, and 0.864 and 0.903 for internalizing problems.

### Additional measures

Additional measures included demographics and background information (e.g., age, gender, parental income), parental mental distress, youth quality of life (KIDS − 10), and youths’ traumatic experiences and symptoms, both assessed by parents.

### Statistical analyses

#### Treatment effects

According to the protocol [30], the data was initially planned to be analyzed using GLM for repeated measures. However, we decided to use a method better suited to handle a situation with lower sample size and higher missing rate, a scenario not anticipated by the protocol. Treatment effects in CBCL were investigated across three time-points, and analyzed using the method described by Mun et al. [43], which is a modification of the latent curve methodology [44]. Rather than describing development as a trajectory throughout the measurement period, we measured change between discrete time points, allowing for a more specific analysis of change across relevant time periods. So, whereas a latent curve model traditionally tests for linear change across time points, the latent change methodology model examines change between discrete significant milestones, that is, intake, post and follow-up. Mun et al. [34] devised three potential modifications of the latent curve model, depending on the time point chosen as reference. In practice, any time point can be chosen as the reference, and in this study, we used intake as the reference for two change components, in line with model 1a in Mun et al. [34]. In this specification of the model, the residual variance at post and follow-up are constrained to zero, to allow identification of individual latent change curves between intake and post, and between intake and follow-up. Thus, change components represent two change scores that can be regressed on the treatment condition and covariates. Consequently, change component 1 describes mean change and variation in change from intake to post, whereas change component 2 describes change from intake to follow-up. To achieve model identification, the residual variance and covariance of post and follow-up measures were constrained to zero. In Mplus, latent curve models allow full information maximum likelihood (FIML) of missing data, which is a key advantage compared to the Generalized Linear Models (GLM) family of models. As a derived statistic from the analysis, we estimated standardized mean change (Cohen’s d) using estimated variance at intake as the denominator. The within-group effects size was obtained by dividing the estimated change (intake-post) and (intake – follow up) on the square root of the estimated intercept baseline variance. Independent and paired samples t-test were used to investigate changes in treatment effects in externalizing and internalizing problems as assessed by BFS and Top Problems, between and within groups, from intake to end of treatment. If the *p*-value from tests were more than 0.05, we concluded that the difference between groups was statistically non-significant. We followed Cohen’s guidelines [29] for interpreting the magnitude of effect sizes (ES); 0.20 signifies a small effect, 0.50 a medium effect, and ES ≥ 0.80 a large effect [45].

## Results

### Attrition analysis

To test for possible selection effects, the randomisation outcome was regressed on known demographic variables such as parent age, parent role, youth gender, family income and education in a multinomial regression model. The randomization outcome was not related to any of the included demographic outcomes (*G*^*2*^ [12] = 2.88, *p* = 0.995). To test for attrition effects, we examined patterns of missing data across intake, post, and follow-up. Four patterns of attrition were contrasted: present at all time- points (i.e., no missing), present at intake and post (i.e., missing at follow-up), present a time 1 only (i.e., missing at post and follow-up), and other missing patterns. To test for selective attrition, the two groups were crossed with patterns of attrition, and we tested for associations. There were no statistically significant associations between group and attrition (*χ2* [3] = 4.47, *p* = 0.21) or between gender and attrition pattern. Neither did CBCL at intake differ across attrition patterns (*F* (3, 77) = 0.432, *p* = 0.735).

### Treatment outcomes CBCL

Table 3 presents the estimated means (*M*), standard deviations (*SD*), and effect sizes (*ES*) of estimated change for externalizing and internalizing problems, as well as the subscales within these problem domains, as measured by the CBCL. The last columns show the effect sizes of within-group latent change, using the pooled intercept intake *SD* as standard. For all means and change components, the null hypothesis was no group difference between the MATCH and TAU condition, and the table includes the significance-level for each test. At intake, the results indicate no statistically significant differences between the youths assigned to the MATCH and TAU conditions with respect to internalizing or externalizing problems, or on CBCL subscales (*p* ≥ 0.37).

**Table 3:** Outcomes within and between treatment groups

	Intake		Post		Follow-up		Intake to post		Intake to follow-up	
Outcome	M	SD	M	SD	M	SD	ES	(95% CI)	ES	(95% CI)
Externalizing										
MATCH	16.90	10.93	12.45	11.02	11.23	10.62	-0.40	(-0.62 to -0.18)	-0.51	(-0.74 to -0.27)
TAU	16.24	11.34	9.99	10.00	8.81	7.99	-0.54	(-0.80 to -0.29)	-0.63	(-0.85 to -0.40)
Group diff	*p* = 0.77		*p* = 0.28		*p* = 0.26		*p* = 0.39		*p* = 0.44	
Internalizing										
MATCH	19.19	10.10	13.79	9.08	14.11	9.85	-0.50	(-0.75 to -0.25)	-0.49	(-0.78 to -0.20)
TAU	19.61	10.22	10.06	6.72	10.16	8.49	-0.84	(-1.12 to -0.56)	-0.79	(-1.18 to -0.40)
Group diff	*p* = 0.84		*p* = 0.04		*p* = 0.16		*p* = 0.06		*p* = 0.19	
Aggressive Behavior										
MATCH	12.51	7.83	9.35	7.95	8.43	7.62	-0.39	(-0.62 to -0.16)	-0.50	(-0.73 to -0.26)
TAU	12.54	8.39	7.84	7.52	6.41	6.08	-0.57	(-0.82 to -0.32)	-0.70	(-0.95 to -0.45)
Group diff	*p* = 0.99		*p* = 0.37		*p* = 0.22		*p* = 0.30		*p* = 0.20	
Rule-Breaking										
MATCH	4.35	3.85	3.18	3.56	2.94	3.47	-0.32	(-0.51 to -0.12)	-0.39	(-0.62 to -0.15)
TAU	3.73	3.29	2.29	2.79	2.40	2.23	-0.42	(-0.70 to -0.13)	-0.36	(-0.59 to -0.14)
Group diff	*p* = 0.37		*p* = 0.19		*p* = 0.40		*p* = 0.58		*p* = 0.89	
Somatic Complaints										
MATCH	4.82	3.85	3.67	3.47	3.22	2.98	-0.30	(-0.54 to -0.06)	-0.43	(-0.70 to -0.16)
TAU	5.36	3.92	2.92	2.01	2.44	2.01	-0.65	(-0.95 to -0.35)	-0.78	(-1.15 to -0.41)
Group diff	*p* = 0.49		*p* = 0.23		*p* = 0.27		*p* = 0.08		*p* = 0.14	
Withdrawn/Depressed										
MATCH	4.21	3.26	3.42	3.12	3.62	3.02	-0.24	(-0.47 to -0.02)	-0.17	(-0.43 to 0.09)
TAU	4.05	3.04	2.33	2.50	2.42	2.67	-0.51	(-0.78 to -0.24)	-0.48	(-0.77 to -0.19)
Group diff	*p* = 0.77		*p* = 0.09		*p* = 0.09		*p* = 0.14		*p* = 0.11	
Anxiety/Depression										
MATCH	10.04	5.39	6.82	4.86	7.19	5.92	-0.56	(-0.82 to -0.30)	-0.50	(-0.80 to -0.19)
TAU	10.04	5.57	4.62	3.36	5.33	5.07	-0.90	(-1.18 to -0.62)	-0.71	(-1.15 to -0.28)
Group diff	*p* = 0.99		*p* = 0.02		*p* = 0.24		*p* = 0.06		*p* = 0.40	

*Note. ES = Effect size, Group diff = *Between-group difference. The null hypothesis was no group difference between the MATCH and TAU condition (H0: Match-Tau = 0) for all between-group comparisons. *n* = 119–108

For both groups the means at post-test and follow-up suggest a substantial decline in externalizing and internalizing problems from intake. In addition, for the associated CBCL subscales, the mean scores declined from intake to post-test and from intake to follow-up, with a statistically significant reductions in internalizing and externalizing problems in both the MATCH and TAU conditions. For the MATCH group, the strongest standardized change was observed for internalizing problems (*d* = 0.50, *SE* = 0.128, *t*= -3.942, *p* < 0.001), in particular for the Anxiety/Depression subscale (*d* = 0.56, *SE* = 0.135, *t* = -4.151, *p* < 0.001). For the TAU group, the effect size was strongest for internalizing problems (*d* = 0.84, *SE* = 0.143, *t* =-5.897, *p* < 0.001), and parallel to the MATCH group, the largest change was observed in the Anxiety/Depression subscale (*d* = 0.90, *SE* = 0.143, *t*=-6.30, *p* < 0.001). Intake to post-test changes and intake to follow-up changes in internalizing and externalizing scores did not differ statistically significant between the MATCH and TAU conditions.

**Fig. 2:**
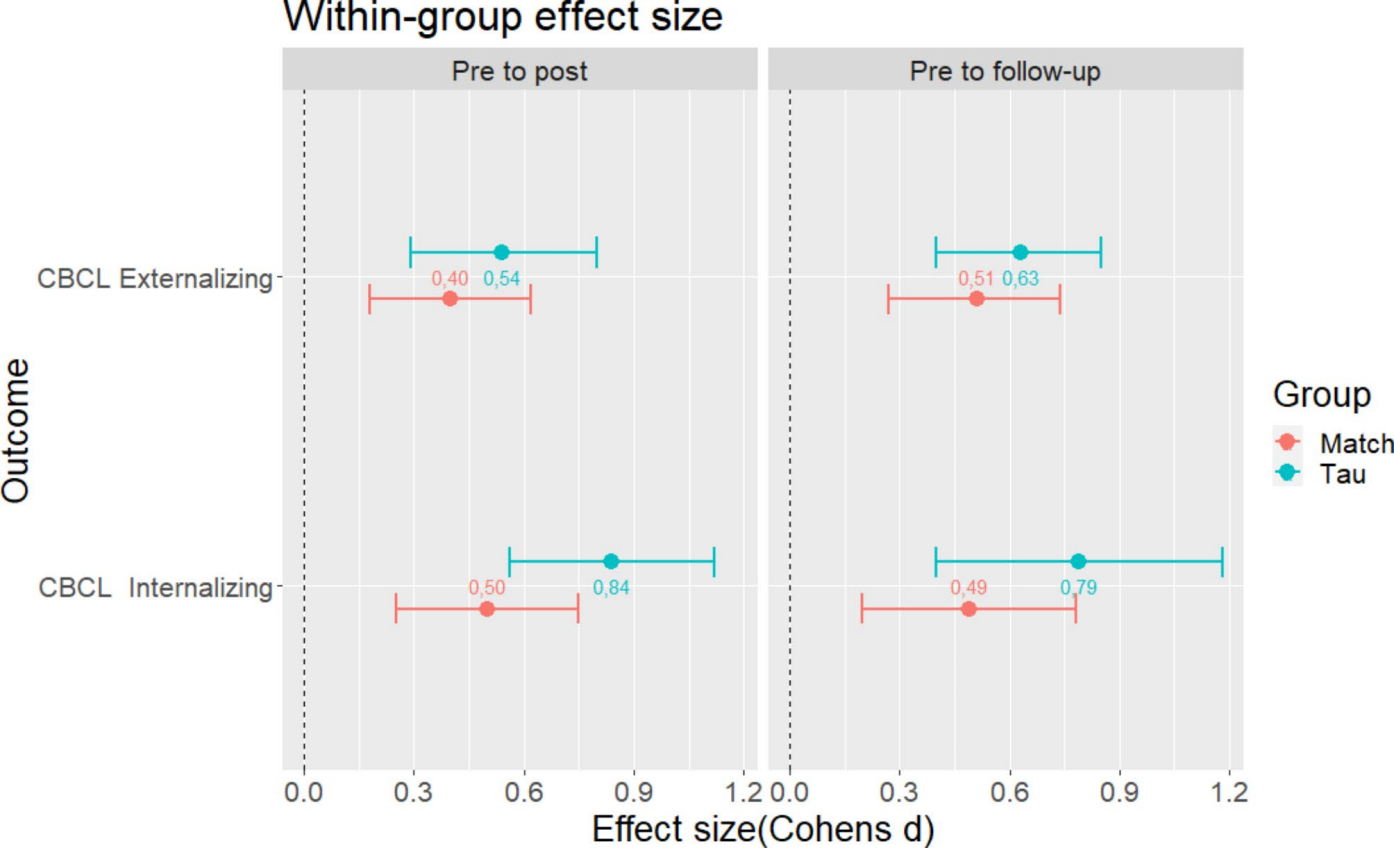
Differences in effect sizes within and between MATCH and TAU groups

We conducted additional analyses adjusting for youth sex, youth age, family income, and parent education. The results showed that the differences in change between MATCH and TAU were not significant, except for the Anxiety/Depression subscale. Specifically, results indicated a significant larger reduction in the Anxiety/Depression score for the TAU group from intake to post-test (Change_MATCH-Change_TAU = 2.16, *t* = 1.965, *p* = 0.049), but not from intake to follow-up (Change_MATCH-Change_TAU = 1.50, *t* = 0.99, *p* = 0.322). Figure 2 shows within-group effect sizes for internalizing and externalizing problems (all significantly different from zero), and between-group confidence intervals, with considerable overlaps for the two groups. As shown, the results indicate moderate to large within-group changes, but no significant group differences in change.

### Treatment outcomes in BFS and TPA

Table 4 shows estimated means (*M*), standard deviations (*SD*), effect sizes (ES) for externalizing and internalizing problems as assessed by BFS and TPA severity ratings. At intake, there were no statistically significant differences in externalizing problems or TPA between the MATCH and TAU group, at intake or post-test. However, a significant difference was found in internalizing problems (*p* = 0.02) at intake, where youths in the MATCH condition exhibited more internalizing problems compared to their counterparts in the TAU condition. The mean scores for internalizing, externalizing, and TPA severity ratings declined significantly from intake to post-test within both groups (*p* < 0.04)

**Table 4:** Outcomes within and between treatment groups

	Intake		Post		Intake to post	
Outcome	M	SD	M	SD	ES	(95% CI)
Externalizing						
MATCH	7.90	6.17	4.16	4.21	-0.63	(-0.35 to -0.90)
TAU	6.48	5.33	4.57	5.14	-0.42	(-0.05 to -0.79)
Group diff	*p* = 0.26		*p* = 0.68		*p* = 0.16	
Internalizing						
MATCH	8.93	5.39	4.47	5.25	-0.77	(-0.50 to -1.04)
TAU	6.33	5.21	4.00	4.32	-0.40	(-0.02 to -0.77)
Groups diff	*p* = 0.02		*p* = 0.67		*p* = 0.07	
Top Problems						
MATCH	8.53	2.30	4.05	2.83	-1.33	(-0.98 to -1.67)
TAU	8.07	2.46	5.15	3.23	-0.96	(-0.49 to -1.42)
Group diff	*p* = 0.38		*p* = 0.11		*p* = 0.07	

Note. ES = Cohen’s effect size, Group diff = Between-group difference. The null hypothesis was no group difference between the MATCH and TAU condition (H_0_: Match-Tau = 0) for all between-group comparisons. *n* = 26– 68

**Fig. 3:**
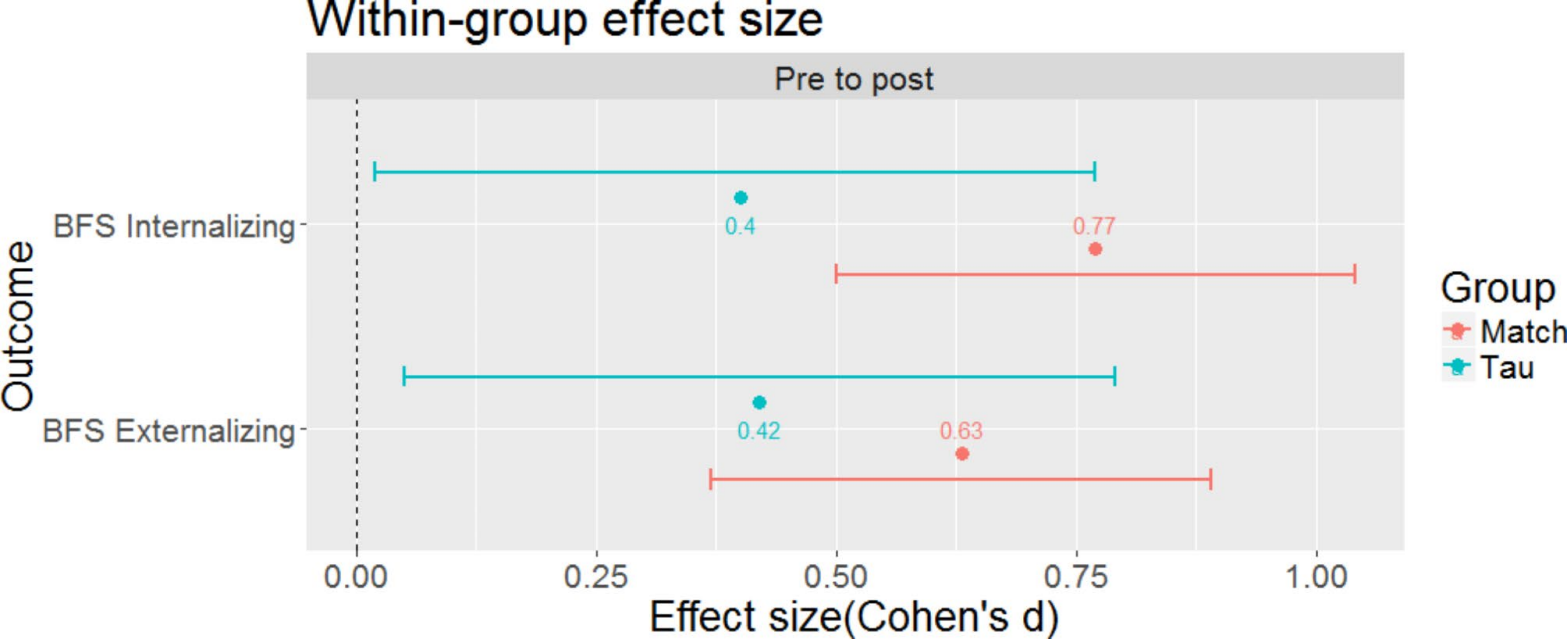
Differences in effect sizes within and between MATCH and TAU groups

From intake to post-test, no significant differences between TAU and MATCH were observed for internalizing and externalizing problems, or TPA severity ratings (*p* ≥ 0.07). Figure 3 shows within-group effect sizes (ES) for internalizing and externalizing problems as assessed by BFS in addition to between-group confidence intervals. As presented, the confidence intervals show considerable overlap, reflecting no significant differences between groups.

### Treatment time

The average number of treatment days from intake to CAMHS to post-test was 333.00 (*SD* = 119.2) for the TAU group and 376.30 (*SD* = 132.76) for the MATCH group. The time between first- day- in-therapy measurement and post-test was *M* = 220.65 days (*SD* = 121.72) for the MATCH group and *M* = 189.33 days (*SD* = 123.33) for the TAU group. The average time from intake or pre-test to follow-up was 682.56 (*SD* = 114.78) for the TAU group and 711.72 (*SD* = 111.05) for the MATCH group. There were no statistically significant differences in treatment duration between the groups.

### Therapist adherence

The results from observational coding of therapist adherence showed that in the MATCH condition, 75.2% of the session content (i.e., 5-minute time segments) was in line with the MATCH coding manual. In the TAU condition, 54.1% of the session content was consistent with the strategies described in the MATCH manual. Paired-samples t-tests indicated that the average number of MATCH strategies per session was higher in the MATCH condition (*M* = 4.5, *SD =* 1.91, range 0–11) than the TAU condition (*M* = 3.9, *SD* = 2.52, range 0–10), with a significant difference between groups (*p* = 0.004). Within each of the four primary protocols (anxiety, depression, conduct problems, and trauma) findings did however reveal non-significant differences in use of MATCH strategies for anxiety (*M*_*MATCH*_ = 4.65, *SD =* 1.86, vs. *M*_TAU_ = 4.21, *SD* = 2.38, *p* = 0*.121*) and depression (*M*_*MATCH*_ = 4.96, *SD =* 2.01, vs. *M*_TAU_ = 5.31, *SD* = 2.57, *p* = 0.572). Among the youths with conduct problems, the use of MATCH strategies was significantly higher in the MATCH condition compared to the TAU condition (*M*_*MATCH*_ = 4.27, *SD =* 1.91, vs. *M*_TAU_ = 1.65, *SD* = 1.53, *p* ≤ 0.001). This was also the case for youths with traumas (*M*_MATCH_ = 3.89, *SD =* 1.78, vs. *M*_TAU_ = 1.33, *SD* = 0.58, *p* = 0.020).

## Discussion

The aim of this study was to assess the effectiveness of MATCH among youths in comparison to TAU, as administered across seven Norwegian CAMHS. Specifically, we aimed to investigate possible treatment effects on parent-reported externalizing and internalizing problems, and parent-identified Top Problems, to determine whether MATCH was more effective in reducing these problems compared to TAU. The results showed overall significant reductions in youth internalizing and externalizing problems, but no significant intervention effects. One year after completion, the effects remained roughly unchanged in both groups for internalizing problems (CBCL), whereas externalizing problems (CBCL) were reduced even further. Average treatment time was comparable for both groups.

In contrast to some previous findings [15, 19, 20], the present study did not find differences in clinical outcomes or treatment duration between the MATCH and TAU conditions. These results were evident from intake to post-test and from intake to follow-up as measured by CBCL, and from intake to post-test as measured by BFS. When considering the five CBCL subdomains, the results confirmed the findings of the broadband measures, indicating no significant differences between MATCH and TAU. The assessments of Top Problems severity showed a trend toward more improvement in MATCH than TAU, but the difference only approached significance (*p* = 0.07). However, due to recruitment rates lower than expected, the present data cannot determine whether MATCH outperforms TAU. The study results do however align with two recent studies [21, 22]. Merry and colleagues [22] found that MATCH and regular practice performed about equally well in reducing youth internalizing and externalizing problems as reported among 206 youths and their parents at post-test, 3 months follow-up, and one year after initial assessment, using two measurement instruments. These results also extended to severity assessments of Top Problems.

Nonetheless, we found that therapists in the MATCH condition showed acceptable adherence to the empirically supported MATCH practices. Based on a sample of therapists from both groups, our coding of therapy sessions indicated that therapists in the MATCH condition performed more EBT practices (about 75% of the session content) than therapists in the TAU condition (about 54% of the session content). This may reflect that the MATCH-therapists increased their use of EBT practices. It may also suggest that therapists in the TAU condition were already practicing a substantial amount of MATCH-related EBT, demonstrating that CBT is a widespread therapeutic method in Norway. In addition, applied EBT competence is a stated goal for future psychologists [46]. In our study, the use of EBT practices was particularly evident in the treatment of anxiety and depression, where no differences were found in the use of MATCH-strategies between the MATCH and TAU therapists. The high level of MATCH EBT content used by the TAU therapists may be one factor explaining why the MATCH and TAU conditions produced comparable outcomes in this study. An analogous hypothesis was suggested in the New Zealand MATCH study, which also showed similar results between MATCH and TAU [22]. Therapists in the TAU condition of the New Zealand trial used a high proportion of the MATCH EBT content (57%), similar to the proportion used by the Norwegian TAU therapists (54%).

It took a considerable amount of time for the Norwegian youths to go from admission to CAMHS and a series of assessments to the first day of therapy. Furthermore, these youths received a greater amount of therapy time (220 days in the MATCH group and 189 days in the TAU group) compared to findings from other studies (e.g., 21, 22). This may partly attributed to the situation in the Norwegian specialist health care system, where lengthy assessment periods before therapy contribute to extended times for treating complex youth problems, exacerbated by an increase in the number of referrals to CAMHS [47]. In addition to the recruitment challenges, several factors could explain the discrepancies between the outcomes of the current study and those showing positive results for MATCH. These factors may encompass culture, randomization procedures, variations in the organisation of youth outpatient clinics, as well as individual characteristics of therapists and youths, all of which can influence study outcomes.

### Strengths and limitations

The present study is one of few to examine MATCH outside the US and the first RCT in a Nordic country. A key strength of this study was that it was implemented in regular clinical CAMHS settings, and thus close to real-life practice and the broader mental health treatment ecosystem [48]. Furthermore, our observational data offered valuable insights into the therapists’ use of empirically supported practices, specifically MATCH-related EBT strategies. Although MATCH therapists were in training, they received substantial supervision and monitoring throughout the therapies to ensure adherence to MATCH procedures. However, MATCH demands both time and efforts from therapists, and the results might have been different if therapists had already completed the training and developed more tacit knowledge that typically comes with years of experience. Additionally, due to the size of the clinics, TAU and MATCH therapists could be working side by side, increasing the risk of spill-over effects between the two conditions.

The study had several limitations. First, we were unable to recruit the number of youths that we had initially planned for [30]. The recruitment challenges impacted the size of the MATCH and TAU groups, reducing the statistical power to detect main effects and potential subgroup effects. Although post hoc power analysis might be of little relevance after results are known, it is relevant to know that a sample size of 121 is able to detect a group by time interaction corresponding to a Cohen’s *d* of 0.26 with a power of 0.80. This indicates that the current study had capacity to identify moderate to strong effects, but not weak effects. Although parent reports are a commonly used source of information regarding youths’ mental health, findings have also revealed discrepancies between parent and child reports on behavioral and emotional problems [49, 50]. This suggests that data from both parents and youths would likely have provided a more accurate and comprehensive understanding of the youths’ problems and needs. The challenge of recruiting families can, in part, be attributed to the high demands placed on the respondents in this study, including lengthy and multiple survey questionnaires. Furthermore, there are indications that MATCH may have been deprioritized in favor of shorter research projects that required fewer resources. It should also be noted that missing data over time was substantial, which may be related to the burden of completing the questionnaires throughout the course of treatment. Some families also dropped out when they discovered they were not assigned to the MATCH group. Although the analysis of dropout mechanisms included relevant predictors, there may still be unmeasured predictors of missing. Missing data could be related to unmeasured variables, such as acute school problems or other sudden adverse conditions. However, in the current study, these potential unmeasured predictors of missing data are unlikely to differ significantly between the two treatment groups. The current study did not include modelling of site and therapist effects, although such effects are likely present. The analytical impact of omitting such effects may be difficult to predict. Previous research suggests a high variability of therapist effects across studies, with and an average intraclass correlation of 0.05 in treatment studies [51]. Extrapolating to the current study it is expected that standard errors might be slightly downward biased. In summary, these limitations suggest that the study’s findings may not be fully generalizable to the broader CAMHS population. Furthermore, our ability to detect intervention effects was limited, so caution is advised when concluding that MATCH had no effects. Additional research into transdiagnostic programs like MATCH in a Nordic context is recommended. These studies should include larger sample sizes, shorter and fewer questionnaires, and assess therapists’ use of EBT practices. Moreover, they should identify and address other obstacles that might lead families to drop out of the study.

### Implications and conclusion

The results of the current study showed overall reductions in youth externalizing and internalizing during treatment, but do not provide evidence that MATCH reduces symptoms of these problems compared to TAU. We did observe that training in MATCH increased the use of EBT components, potentially making it more time-efficient than training in single interventions for homogenous disorders. Consequently, decision-makers should consider the time and resource costs associated with implementing single disorder treatments, particularly when transdiagnostic approaches are available.

## Electronic supplementary material

Below is the link to the electronic supplementary material.

Supplementary Material 1

## Data Availability

No datasets were generated or analysed during the current study.

## Abbreviations

NCCBD: The Norwegian Center for Child Behavioral Development
MATCH: The Modular Approach to Therapy for Youths with Anxiety, Depression, Trauma and Conduct Problems
TAU: Treatment as Usual
EBT: Evidence-based treatment
CAMHS: Child and Adolescent Mental Health Clinics
TF CBT: Trauma-Focused Cognitive Behavioral Therapy
PMTO: Parent Management Training the Oregon Model
RCT: Randomized Controlled Trial
TPA: Top Problems Assessment
PATH: Program Assessment in Therapy
CBCL: Child Behavior Checklist
ASEBA: Achenbach System of Empirically Based Assessment
BFS: Behavior and Feelings Survey
FIML: Full Information Maximum Likelihood
GLM: Generalized Linear Models
ES: Effect Size
SD: Standard Deviation
